# Injury shortens life expectancy in ants and affects some risk-related decisions of workers

**DOI:** 10.1007/s10071-023-01810-0

**Published:** 2023-07-14

**Authors:** Filip Turza, Krzysztof Miler

**Affiliations:** 1 Doctoral School of Exact and Natural Sciences, Jagiellonian University, Prof. S. Łojasiewicza 11, 30-348 Kraków, Poland; 2 Institute of Environmental Sciences, Faculty of Biology, Jagiellonian University, Gronostajowa 7, 30-387 Kraków, Poland; 3 Institute of Systematics and Evolution of Animals, Polish Academy of Sciences, Sławkowska 17, 31-016 Kraków, Poland

**Keywords:** Aggression, Ants, *Formica cinerea*, Rescue behavior, Survival

## Abstract

The *Formica cinerea* ants are known to be highly territorial and aggressively defend their nest and foraging areas against other ants. During the foraging, workers engage in large-scale battles with other colonies of ants and injuries often occur in the process. Such injuries open the body up to pathologies and can lead to costs expressed in lower survival. Here, we addressed the significance of injury in dictating decisions related to engagement in risky behavior in ants (i.e., rescue and aggression). We manipulated the life expectancies of *F. cinerea* workers by injury and found that the survival of injured workers was shorter compared to the intact individuals. Furthermore, we found that injured workers discriminated between the intact and injured nestmates and showed more rescue behavior toward intact individuals. These rescue actions were expressed as digging around the trapped ant in need of rescue, pulling at its body parts, transporting the sand covering it, and biting the thread entrapping it. In turn, intact and injured workers showed similar and high levels of aggression toward heterospecifics. Our findings highlight the role of behavioral context in the studies devoted to the decision-making processes among social insects and the importance of life expectancy in their behavioral patterns.

**Supplementary Information:**

The online version contains supplementary material available at 10.1007/s10071-023-01810-0.

## Introduction

Injuries result from various factors, such as fights over resources or failed predation. Ants are commonly characterized by non-lethal injuries (Gilad et al. 2022). For instance, slave-making ants (*Formica sanguinea*) inflict wounds on their hosts, such as the removal of one or more legs or antennae (Hölldobler and Wilson 1990). Confrontation with termites leads to similar injuries in termite-eating ants (*Megaponera analis*) (Frank et al. 2017). These injuries might have various costs and create a risk of higher mortality by opening the body up to infection, desiccation, function impairment, and/or other pathologies.

Not only in ants but also in many other species of social insects, task engagement is dictated by life expectancy (Woyciechowski and Kozłowski 1998). Indeed, young and fit workers choose tasks inside the nest and switch to foraging outside when they get older (Hölldobler and Wilson 1990). This is accompanied by many physiological changes, e.g., altered protein and hormone levels (Hartmann et al. 2020). Behavioral variation can be observed even within groups of workers belonging to the same sub-caste, as in termites, in which older soldiers are more risk-prone than younger soldiers (Yanagihara et al. 2018, see also Moroń et al. 2008). Here, we decided to test the importance of life expectancy in the context of risky rescue behavior, in which individuals act to free a nestmate from danger after it signals distress (Czechowski et al. 2002; Miler and Kuszewska 2017). The literature refers to various contexts where ants show this common behavior (Miler and Turza 2021). However, there is a considerable unexplained variation in rescue occurrence on both between-species and between-individuals levels (Miler et al. 2017a; Andras et al. 2020). In our study, we examined how an injury might affect survival and the decision-making process in terms of rescue engagement in *Formica cinerea* ants. To assess context-specificity, we checked also another risky behavior, aggression. As our model species is vulnerable to attack from predators such as antlions, and other ants, it has a high number of injured individuals and shows marked levels of both rescue and aggression (Czechowski et al. 2002; Miler 2016).

We hypothesize that (1) injured workers would be characterized by lower life expectancy than intact individuals. This would be in line with studies that utilized experimental groups in which life expectancy was artificially shortened (e.g., Moroń et al. 2012). To add ecological relevance, we compare the survival of naturally and experimentally injured workers. Second, we hypothesize that (2) injured workers would be more responsive in terms of rescue and aggression. Injured workers could be less valuable to the colony due to their impaired performance, susceptibility to predation/infection, and shorter life expectancy, so they might show a lower threshold for engaging in more risky tasks than intact workers (Tofilski 2009). This is reasonable to expect based on the age-dependent changes in behavior among social insects (Woyciechowski and Kozłowski 1998; Hartmann et al. 2020). Third, we hypothesize that (3) rescue would be directed less toward injured than intact workers. Since rescue occurs to benefit the colony, saving soon-to-die individuals should be counterproductive (Miler et al. 2017b).

## Materials and methods

Ant foragers were collected near Klucze (Poland, 50°21′22″N 19°31′03″E) from three colonies, with a week-long break between colonies, in the summer of 2022. Ants were transported to the laboratory, kept in containers (40 × 30 × 10 cm) at a constant temperature of 24 °C, 40–60% RH, and 12:12 day/night cycle with ad libitum access to water and 10% sucrose solution.

In the survival experiment, three groups of workers per colony, counting 30 individuals each, were created: a control group (C), a naturally injured group (N), and an experimentally injured group (E). The control group contained only intact individuals. The naturally injured group comprised individuals with at least a part of some extremity (antenna or leg) missing. In the experimentally injured group, each individual had the left or right hind leg removed at the femur using microscissors. All groups of ants were kept in the experimental boxes (18 × 15 × 7 cm) separate for each group and colony for the standard survival rate observation (Miler 2016).

In the behavioral experiment, the analogical control group of intact individuals (C) and experimentally injured group of workers (E) was placed in a shared container and tested for their rescue behavior and aggression toward nestmates and heterospecifics, respectively. Rescue behavior was tested using four standard dyadic rescue behavior tests (CC vs. CE vs. EC vs. EE) (Nowbahari et al. 2009), where a forager from group C or E was captured and placed in the middle of a sand-filled box, partly covered with sand, and a potential rescuer from the same or opposite group was placed in the arena. Aggression behavior was tested using two types of dyadic aggression tests (HC vs. HE), where a heterospecific individual (*Lasius niger*) was placed as the victim, and the procedure was the same as for rescue behavior tests. All behaviors were analyzed using standard rescue and aggression behavioral categories (Nowbahari et al. 2009) (see Appendix A) in terms of occurrence (1/0) and duration (s).

Statistical analyses were performed using R (R Core Team 2023). To analyze survival, we used a mixed-effects Cox proportional hazards model (coxme package; Therneau 2020) with a random factor “colony” and a fixed factor “mortality group” (C vs. N vs. E). Data for ants that remained alive after 50 days were censored. To compare rescue occurrence, we used a generalized linear mixed model (lme4 package; Bates et al. 2015) with a binomial residual distribution, logit link function and included a random factor “colony” and fixed factors “victim type” (C vs. E) and “rescuer type” (C vs. E), and their interaction. To compare the duration of rescue, we used a mixed-effects Cox proportional hazards model (coxme package; Therneau 2020) with the same factors. To compare aggression occurrence, we used a binomial generalized linear mixed model (lme4 package; Bates et al. 2015) with a random factor “colony” and a fixed factor “aggressive group” (C vs. E). To compare the duration of aggression, we used the mixed-effects Cox model (coxme package; Therneau 2020), with the same factors. In the Cox models, the data for tests during which rescue and aggression were interrupted at the end of the recording (i.e., 5 min) was censored.

## Results and discussion

**Fig. 1:**
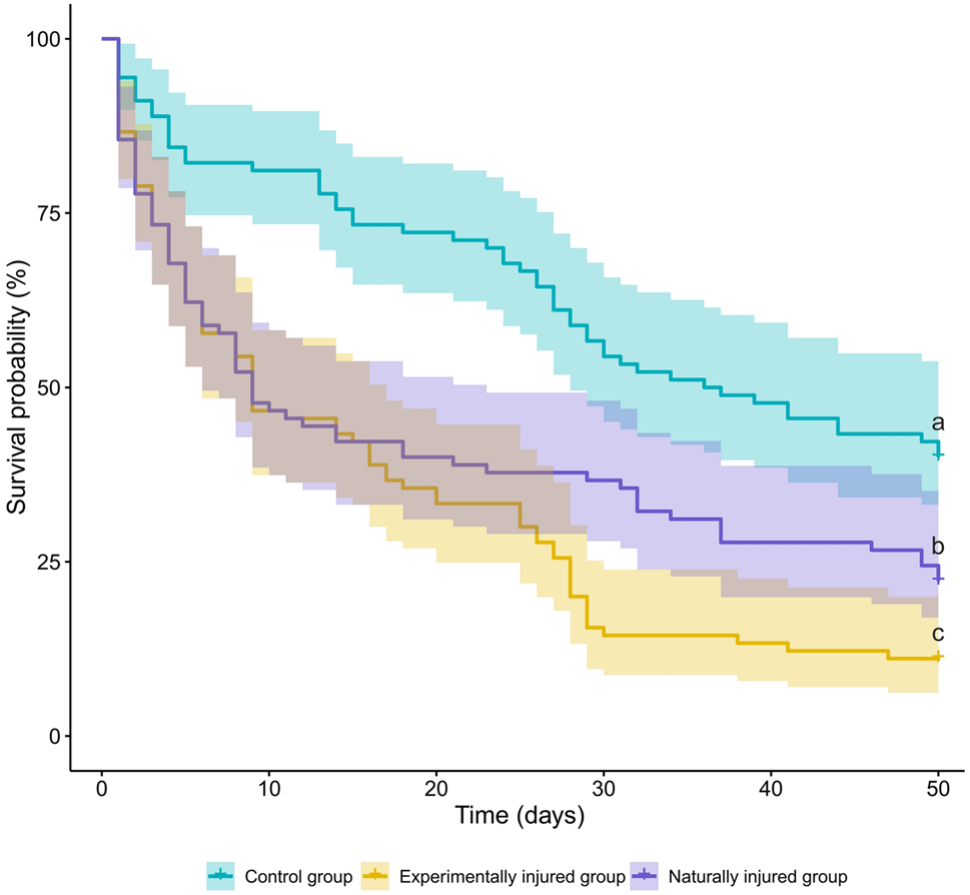
Survival curves for different groups of foragers in *F. cinerea*. Shading indicates 95% confidence intervals. Small letters above crosses indicate significance yielded by between group comparisons (with *p* < 0.001)

In agreement with our first hypothesis, we observed lower survival of injured foragers (as shown by the comparison of the survival in groups C and E, *z* = 5.490, *p* < 0.001; Fig. 1). Although this result might seem intuitive, it is crucial to demonstrate. At times, surprising patterns related to life expectancy are revealed (e.g., some parasitised ants show prolonged lifespans, see Beros et al. 2021). Notably, we also observed a significant difference in the survival of the experimentally treated and naturally injured group (*z* = 2.240, *p* < 0.001; Fig. 1) (see Appendix B for full results). This result might stem from differences between conditions in which the ants were injured. Indeed, a laboratory setting with abnormal colony interactions, e.g., without wound grooming by healthy individuals inside the nest (Frank et al. 2018), was likely affecting the mortality rate. In any case, we confirm treatment by injury is effective in reducing life expectancy. However, further research should focus on the possibility of creating more natural conditions in the laboratory, e.g., by keeping experimentally treated ants with access to the full colony, and so ensuring the presence of natural interactions between individuals of different castes and/or health status.

**Fig. 2:**
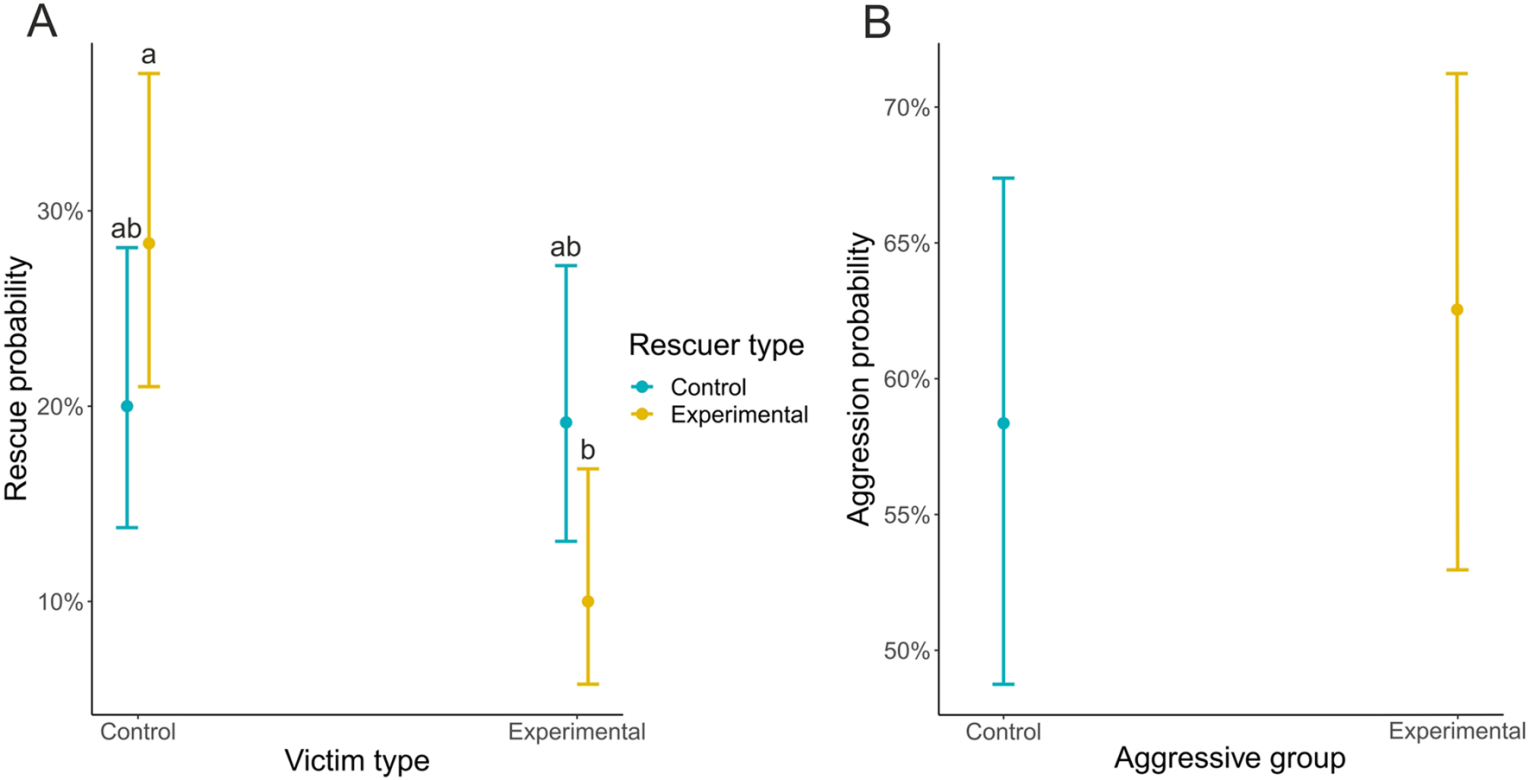
Probability of rescue (**A**) and aggression (**B**) in *F. cinerea* foragers. Dots represent model predictions and whiskers indicate 95% confidence intervals. Small letters above upper whiskers indicate significance yielded by post hoc Tukey comparisons

That said, our second and third hypotheses were only partially confirmed. Rescue probability differed between tests (victim type: *χ*^2^ = 0.027, *p* = 0.871; rescuer type: *χ*^2^ = 2.256, *p* = 0.133; interaction: *χ*^2^ = 6.177, *p* = 0.013; Fig. 2A). Specifically, injured workers took part in rescue actions more likely toward intact nestmates. Regarding the aggression directed toward heterospecific ants, there was no difference between the intact and injured groups (*χ*^2^ = 0.437, *p* = 0.500; Fig. 2B). In both rescue and aggression, the total duration of these behaviors was similar in all tests (see Appendix B for full results). A higher probability of providing help to individuals with higher life expectancies rather than soon-to-die individuals illustrates the adaptive significance of rescue (Miler 2016). However, why only injured workers discriminated between intact and injured individuals remains unclear. Although the latter likely differ in cuticular hydrocarbon profiles and/or volatile pheromones from normal individuals (Csata et al. 2023), a lack of discrimination between nestmates that differ in life expectancies is already reported in the literature (Leclerc and Detrain 2016). Regarding aggression, previous research suggests that some ants with shortened life expectancies increase their level of aggression toward heterospecific ants (Bos et al. 2012). Why is the decision to attack not related to life expectancy in our species unclear, but possibly, *F. cinerea* is too territorial and aggressive to show dependence on life expectancy in this context (Czechowski et al. 2002). It can be worthwhile to compare aggressive tendencies in normal and injured workers of several species differing in their competitiveness.

Our results show that injury in *F. cinerea* foragers leads to a survival cost, and this cost affects rescue but not aggressive behavior. Indeed, injured workers were more likely to perform risky rescue actions toward intact individuals, while this type of discrimination between nestmates did not occur in intact ants. At the same time, aggression toward heterospecifics was similar in both injured and intact workers. This supports a growing number of studies that indicate that ants are highly sensitive to the behavioral context (Duhoo et al. 2017; Turza and Miler 2022). In the case of our study, we demonstrate diverging responses of ants to decreased life expectancy in the context of rescue and aggression. So far, little research attention was placed on the continued role of injured individuals in the colony (Tofilski et al. 2008; Gilad et al. 2022) and we encourage further studies on the role of decreased life expectancy on behavior of social insects. From a larger perspective, to fully describe the effect of life expectancy on behavior, experiments need to include this and other factors, such as aspects of ecology, body size, and/or personality, together (Hollis and Nowbahari 2013; Okrutniak et al. 2020; Maák et al. 2021).

## Supplementary Information

Below is the link to the electronic supplementary material.

Supplementary file1 (PDF 212 KB)

## Data Availability

All data generated and analyzed during the current study are available in the RODBUK repository, [https://doi.org/10.57903/UJ/OAUSU5].
