# Supplementary material for: Injury shortens life expectancy in ants and affects some risk-related decisions of workers
Source: Anim Cogn. 2023 Jul 14;26(5):1643–7. doi: 10.1007/s10071-023-01810-0 (PMC10442280; doi:10.1007/s10071-023-01810-0)
Supplement: Supplementary file 1 — Supplementary file1 (PDF 212 KB) [file 10071_2023_1810_MOESM1_ESM.pdf]

Injury shortens life expectancy in ants and affects some risk-related decisions of workers

Filip Turza, Krzysztof Miler

Correspondence:

Filip Turza, e-mail: filip.turza@uj.edu.pl

Krzysztof Miler, e-mail: miler@isez.pan.krakow.pl

Supplementary Materials

Appendix A. Operational definitions of the behavioural categories of rescue and aggression.

Appendix B. Full results of the Cox mixed-effects models conducted in the study.

Appendix A

**Rescue**	**Operational definition**
Digging	The ant stands in front of the victim and flicks sand backward, using legs
Pulling	The ant grabs any part of the body of the victim and drags it backward, using mandibles
Sand transport	The ant picks up one or more sand particles, covering the filter paper or the victim, and moves it away, using mandibles
Thread biting	The ant bites and tugs on the nylon thread holding the victim, using mandibles
**Aggression**	**Operational definition**
Biting	The ant bites any part of the body of the victim, using mandibles and flexes its gaster underneath its body
Formic acid projection	The ant flexes its gaster underneath its body and projects formic acid in the direction of the victim
Threatening	The ant opens mandibles to their maximally widest position in the direction of the victim

Table 1. Definitions of rescue and aggression categories used in the study.

Appendix B

	exp(coef)	se(coef)	z	P-value
**C vs. E**	2.692	0.181	5.490	< 0.001
**C vs. N**	1.849	0.182	3.390	< 0.001
**E vs. N**	0.687	0.167	2.240	< 0.001

Table 1. Full results of the survival analysis conducted using the Cox mixed-effects model by maximum likelihood for the 3 treatments: a control group of intact individuals (C), a group comprised of individuals with at least a part of some extremity (antenna or leg) missing (N), and a group treated experimentally in which intact individuals had one of their hindlegs removed at the femur (E).

	χ^2	P-value
**Victim type**	0.376	0.540
**Rescuer type**	0.050	0.823
**Victim type × rescuer type**	1.009	0.315

Table 2. Full results of the rescue duration analysis conducted using the Cox mixed-effects model by maximum likelihood with a random factor “colony” and fixed factors “victim type” and “rescuer type” and their interaction. Data for tests during which rescue behaviour was interrupted at the end of the recording (i.e., 5 minutes) was censored.

	χ^2	**P-value**
**Aggressive group**	2.374	0.123

Table 3. Full results of the aggression duration analysis using the Cox mixed-effects model by maximum likelihood with a random factor "colony" and a fixed factor "aggressive group". Data for tests during which aggression behaviour was interrupted at the end of the recording (i.e., 5 minutes) was censored.
